# Chitosan nanoencapsulation of *Turbinaria triquetra* metabolites in the management of podocyturia in nephrotoxic rats

**DOI:** 10.1038/s41598-024-82463-y

**Published:** 2025-01-03

**Authors:** Abdullah Abdel-Aal, Abdel-Aziz A. F., Zakaria El-khayat, Nadia Mohamed, Merit Rostom, Emad Tolba, Nihal Galal El-Din Shams El-Din, Walaa S. A. Mettwally, Abdelhamid Aly Hamdy

**Affiliations:** 1 Biochemistry Division, Faculty of Science, Mansoura University, Mansoura, Egypt; 2 Medical Biochemistry Department, National Research Centre, Dokki, Giza Egypt; 3 Academy of Scientific Research and Technology, ASRT, Cairo, Egypt; 4 Polymers and Pigments Department, National Research Centre, 33 El Bohouth St, Dokki, Cairo, 12311 Egypt; 5 National Institute of Oceanography and Fisheries (NIOF), Cairo, Egypt; 6 Chemistry of Natural and Microbial Products Department, Pharmaceutical and Drug Industries Research Institute , National Research Centre, Dokki, Cairo, 12622 Egypt

**Keywords:** Brown alga, ***Turbinaria Triquetra***, Polysaccharide, Chitosan nanoparticles, Cisplatin, Nephrotoxicity, Podocyturia, Biochemistry, Biological techniques

## Abstract

Cisplatin is a chemotherapeutic drug, which exhibits undesirable side effects. Chitosan nanoparticles are promising for drug delivery. The aim of this study was to determine the effect of the brown alga *Turbinaria triquetra* ethyl acetate fraction and polysaccharides, either loaded on chitosan nanoparticles or free, against podocyturia and cisplatin nephrotoxicity in rats. Sixty-six male rats were distributed into 11 equal groups: untreated control, chitosan (CSNPs), ethyl acetate fraction (EAE), polysaccharide (PS), EAE loaded on chitosan nanoparticles (EAE-CSNPs), PS loaded on chitosan nanoparticles (PS-CSNPs), Cisplatin or cis-diamminedichloroplatinum(II) (CDDP), CDDP + EAE, CDDP + PS, CDDP + EAE-CSNPs, and CDDP + PS-CSNPs. Serum urea, creatinine, creatinine clearance, renal malondialdehyde, nitric oxide, paraoxonase 1, renal nephrin, and podocin, and their renal mRNA gene expressions, as well as urinary nephrin and podocin were determined. The results indicated that the ethyl acetate fraction and polysaccharides, either free or loaded, efficiently attenuated podocyturia and cisplatin nephrotoxicity compared to the Cis group. However, the improvement was higher in the nephrotoxic groups treated with EAE-CSNPs and PS-CSNPs. The current study revealed that chitosan nanoencapsulation showed ameliorative effects against podocyturia and cisplatin nephrotoxicity in rats compared to free extracts, offering a new therapeutic strategy for attenuating podocyturia and CDDP-induced nephrotoxicity.

## Introduction

Cisplatin (CDDP) remains one of the best chemotherapeutic drugs usually used for the treatment of many tumors, even with its harmful side effects^1^. One of the complications of CDDP treatment is nephrotoxicity, which may be attributed to several mechanisms such as DNA damage, inhibition of protein synthesis, mitochondrial dysfunction, cell membrane peroxidation, and oxidative stress that can eventually lead to damage of the renal tissue^2^. Oxidative stress can be defined as any condition in which there is a significant imbalance between the antioxidant system and reactive oxygen species (ROS)^3^. Two main kinds of chemicals that make up ROS are free radicals, such as nitric oxide and hydroxyl radicals, and non-reactive radicals, such as hypochlorous acid and aldehydes. Under physiological conditions, both intracellular and extracellular environments have antioxidants that are enzymatic, like Paraoxonase 1, and non-enzymatic like vitamin C that are crucial for the cellular response to deal with oxidative stress and detoxify ROS^3^.

Oxidative/nitrosative stress, inflammation, and apoptosis roles have been described in many studies as prominent factors in mediating many pathological alterations, in response to toxic agents or in disease states^4^; however, it is not well characterized whether there is an interplay between these three factors or any combination of them, in addition to their sole effect, in mediating the harmful mechanisms of pathological alterations particularly in the heart, liver, and kidney. These harmful mechanisms might be identified directly via exposure to various insults or indirectly via evaluating the mechanism(s) of the prevention of these pathological alterations or by both methods^4^.

The kidney is one of the vital organs that maintains homeostasis in the human body and excretes waste products through its functional units; the nephrons, which consist of glomeruli as well as proximal and distal tubules. Each glomerulus exerts its vital role via the glomerular filtration barrier (GFB), which consists of three layers: endothelial cells, glomerular basement membrane (GBM), and podocytes^5^. The genetic investigations of humans have indicated that podocytes play a crucial role in keeping GFB function. Additionally, it has been demonstrated that any impairment of podocytes leads to the formation of fibrous lesions within the glomerulus. Moreover, there are a multitude of genes that undergo mutation or possess variations that contribute to an elevated susceptibility to disease and are expressed by podocytes. Consequently, several of these genes have been examined as potential targets for therapeutic interventions^6^. Two of the structural proteins that constitute podocytes are nephrin and podocin that their interaction is important for healthy podocytes and normal GFB. Nephrin, a 180 kDa protein, is a key element of the slit diaphragm and is found on the podocyte foot process surface^7^. Podocin is a membrane-associated 42 kDa protein with a hairpin shape, which causes both of its terminal ends to be found in the cytoplasm^8^. Patients with various glomerulopathies have had urine samples positive for nephrin and podocin proteins, which have the potential to serve as diagnostically meaningful biomarkers of podocyte damage^8,9^. The presence of nephrin, podocin, or other proteins related to podocytes in the urine can be defined as podocyturia^10^.

In the last decade, a great interest has been established in extracting bioactive compounds with antioxidant properties from algae^11^. The brown alga *Turbinaria triquetra* (J.Agardh) Kützing (*T. triquetra)*comprises primary algal metabolites like polysaccharides including alginate, fucoidan, laminaran, and mannitol, as well as secondary metabolites like pigments, phenolics, sterols, terpenes, halogenated compounds, and small peptides, among other bioactive compounds^12^. Ethyl acetate was selected as an organic solvent for the extraction of high and low molecular weight phenolic compounds^13^.

In the last 30 years, materials at the nanostructured materials have become recognized as a novel instrument with a variety of special uses in medical imaging, diagnosis, and even treatment. Since then, there have been many examples of how the application of nanomaterials in medicine, or nanomedicine, has advanced to meet hitherto unmet medical requirements. Because of their physicochemical properties, pharmacokinetics, and biodistribution, which can be tuned, better drug delivery systems have been developed. These nanosystems aim to reduce side effects and increase therapeutic efficacy by primarily targeting malignant (or diseased) areas rather than healthy ones. Indeed, polymeric nanoparticles (NPs) can be used to deliver medicinal plants extracts to specified tissue or organs without harming the healthy surrounding tissues^14^. Among these nanoparticles are chitosan nanoparticles (CSNPs) that have relative non-toxicity, biodegradability, biocompatibility, bioadhesive, permeability-enhancing, and cationic properties^15^.

Thus, the aim of the present research was to detect the effect of both the ethyl acetate fraction (EAE) and the polysaccharide (PS) extracted from *T. triquetra*, either in free form or loaded on chitosan nanoparticles against CDDP-induced nephrotoxicity and podocyturia in rats. It was our intention in this special study to invite new insights and to shed a light on very interesting topic that would identify new therapeutic targets for nephrotoxicity, in the context of oxidative stress, inflammation, and apoptosis, and to tackle them in hope of better treatments.

## Results

### Determination of antioxidant activity

The scavenger percentage (%) and half- maximum inhibitory concentration (IC50) of EAE were calculated and illustrated in Table 1. The antioxidant activity of EAE was compared with our previously published activity of PS by Abdel-Aal et al.^16^ (Table 1). The results revealed scavenger percentage as follows; EAE > PS.

**Table 1:** Scavenger percentage (%) and IC50 of the samples.

Sample (1 mg/ml)	Scavenger percentage (%)	IC50 (mg/ml)
EAE	30	1.67
PS	24	2.08
Control (ascorbic acid)	62	0.80

### Determination of total phenolic content

**Fig. 1:**
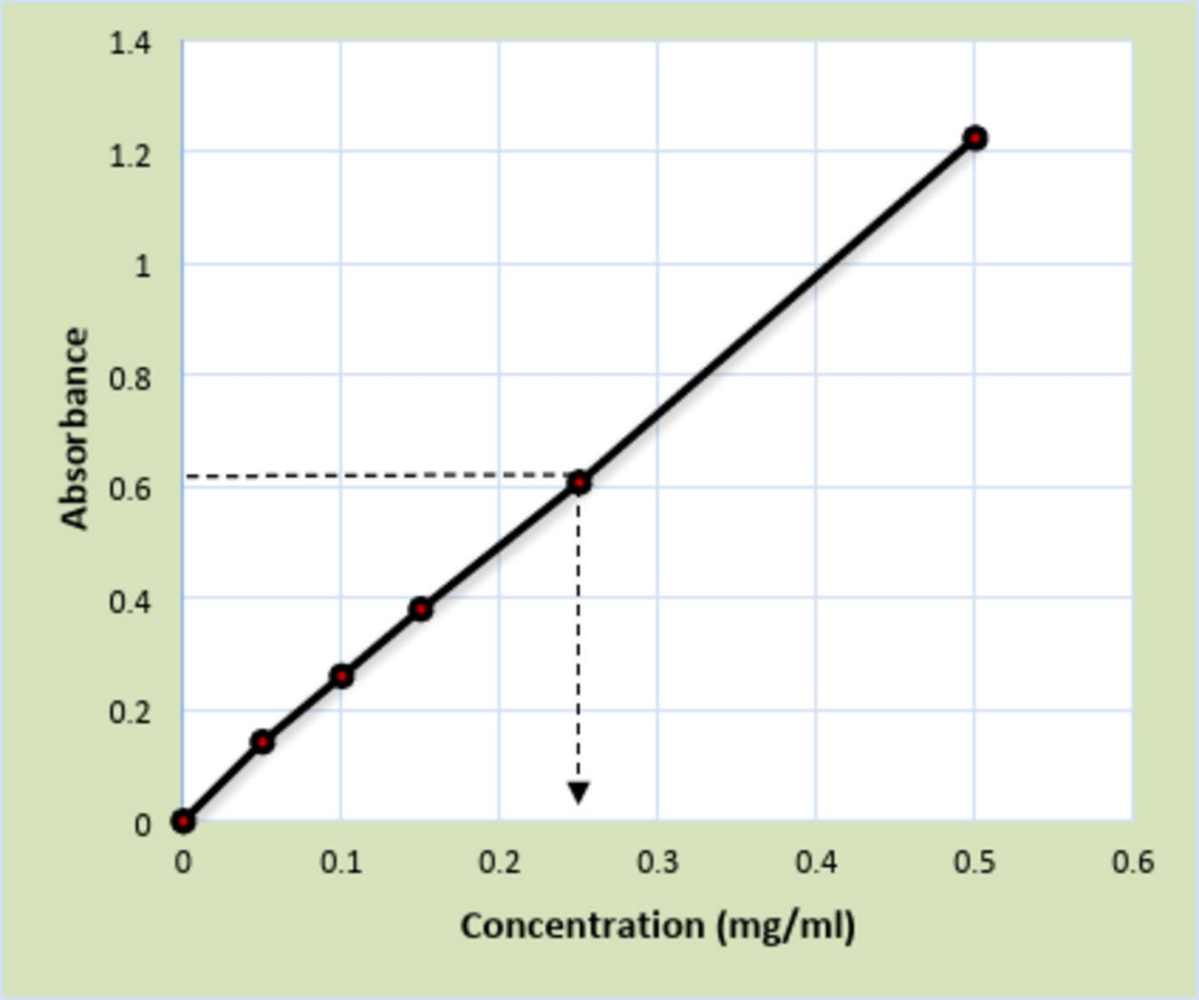
Gallic acid standard curve.

The assay of (7 mg/ml) of ethyl acetate fraction (EAE) revealed absorbance at 0.6, which was equivalent to 0.25 mg/ml on the gallic acid standard curve (Fig. 1). Hence, the total content of phenolics in EAE was calculated and determined (35.7 mg GAE/ g of dry fraction).

### Microstructure characterizations

**Fig. 2:**
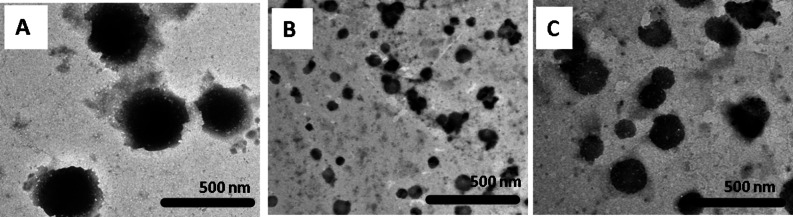
TEM micrographs of (A) CSNPs, (B) EAE-CSNPs and (C) PS-CSNPs.

In this study, EAE and PS-loaded chitosan nanoparticles were prepared by the ionic gelation approach. The morphology of the obtained NPs is shown in Fig. 2. However, the neat CSNPs and EAE (or) PS-loaded CSNPs revealed a spherical shape. The average particle sizes of the fabricated nanoparticles were between 270 ± 82 nm for CSNPs and 148 ± 35 nm for PS-CSNPs, while the average particle sizes for EAE-CSNPs were around 65 ± 17 nm. Moreover, DLS and zeta potential of the measured samples are represented in Table 2. The DLS results demonstrated larger particle sizes than those observed through TEM. In the present study, the formed nanoparticles revealed a positively charged surface as follows: EAE-CSNPs > PS-CSNPs > CSNPs.

**Table 2:** DLS measurements: average size, zeta potential and polydispersity index (PDI) of CSNPs, EAE-CSNPs and PS-CSNPs.

Nanoparticles	Average size (nm)	Zeta potential(mV)	PDI
**CSNPs**	490.1 ± 31.4	22.2 ± 1.2	0.319
**EAE-CSNPs**	125.3 ± 22.8	36.8 ± 2.4	0.151
**PS-CSNPs**	360.5 ± 33.7	33.4 ± 1.8	0.223

### Biochemical assays

#### Effect of treatments on serum urea, creatinine and creatinine clearance***(***Fig. 3) and oxidative stress markers (renal MDA, NO and PON 1) (Fig. 4)

**Fig. 3:**
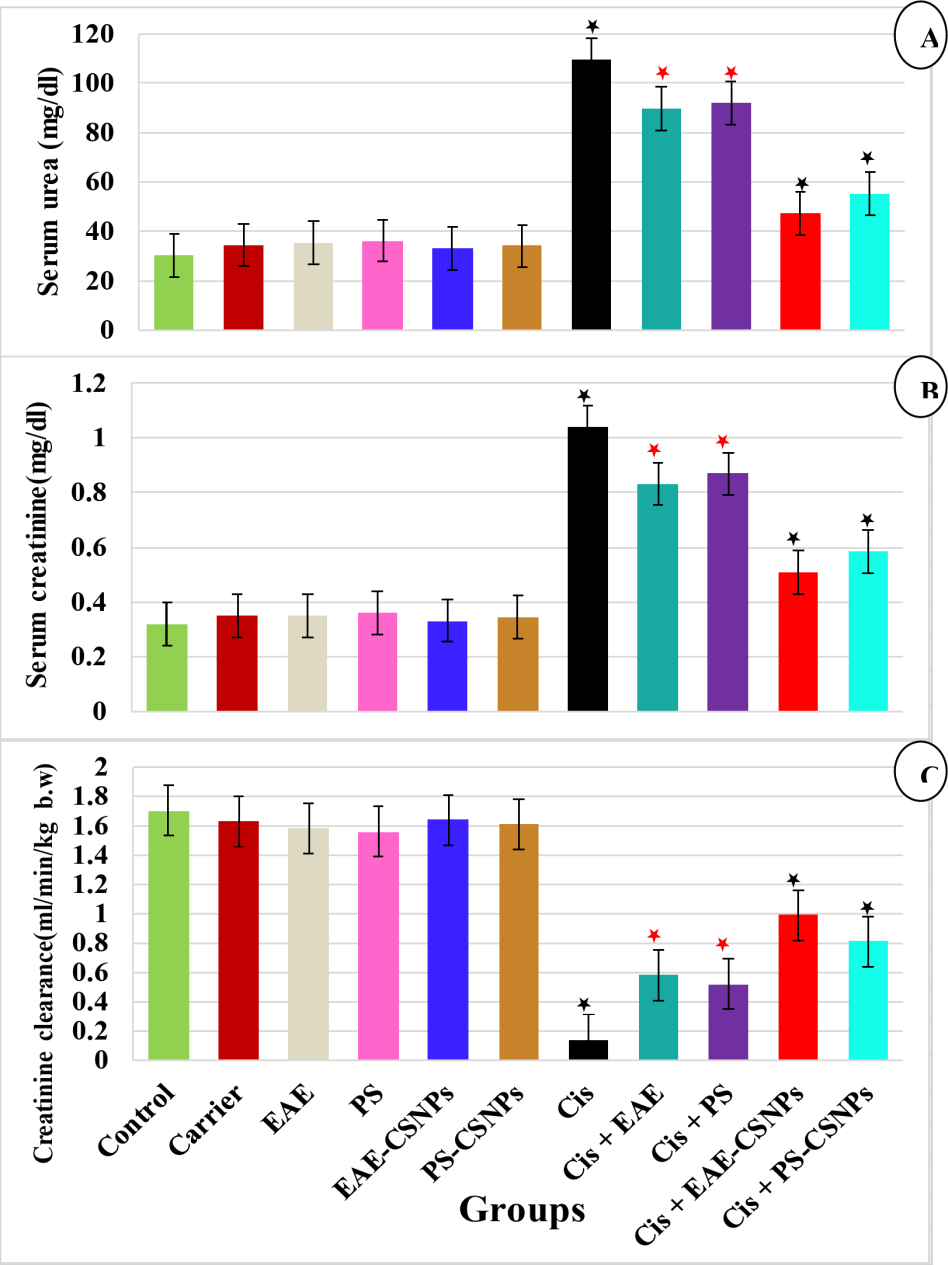
Mean value of (A) Serum urea (mg/dl), (B) Serum creatinine (mg/dl) and (C) Creatinine clearance (ml/min/kg bw) in different groups. There was no significance difference between the six first groups (control-PS-CSNPs). There was significance difference between Cis group and Cis + EAE and Cis + PS (*p* < 0.05*), while there was high significance between Cis group and Cis + EAE-CSNPs and Cis + PS-CSNPs (*p* < 0.001*). Error bars represent standard error.

**Fig. 4:**
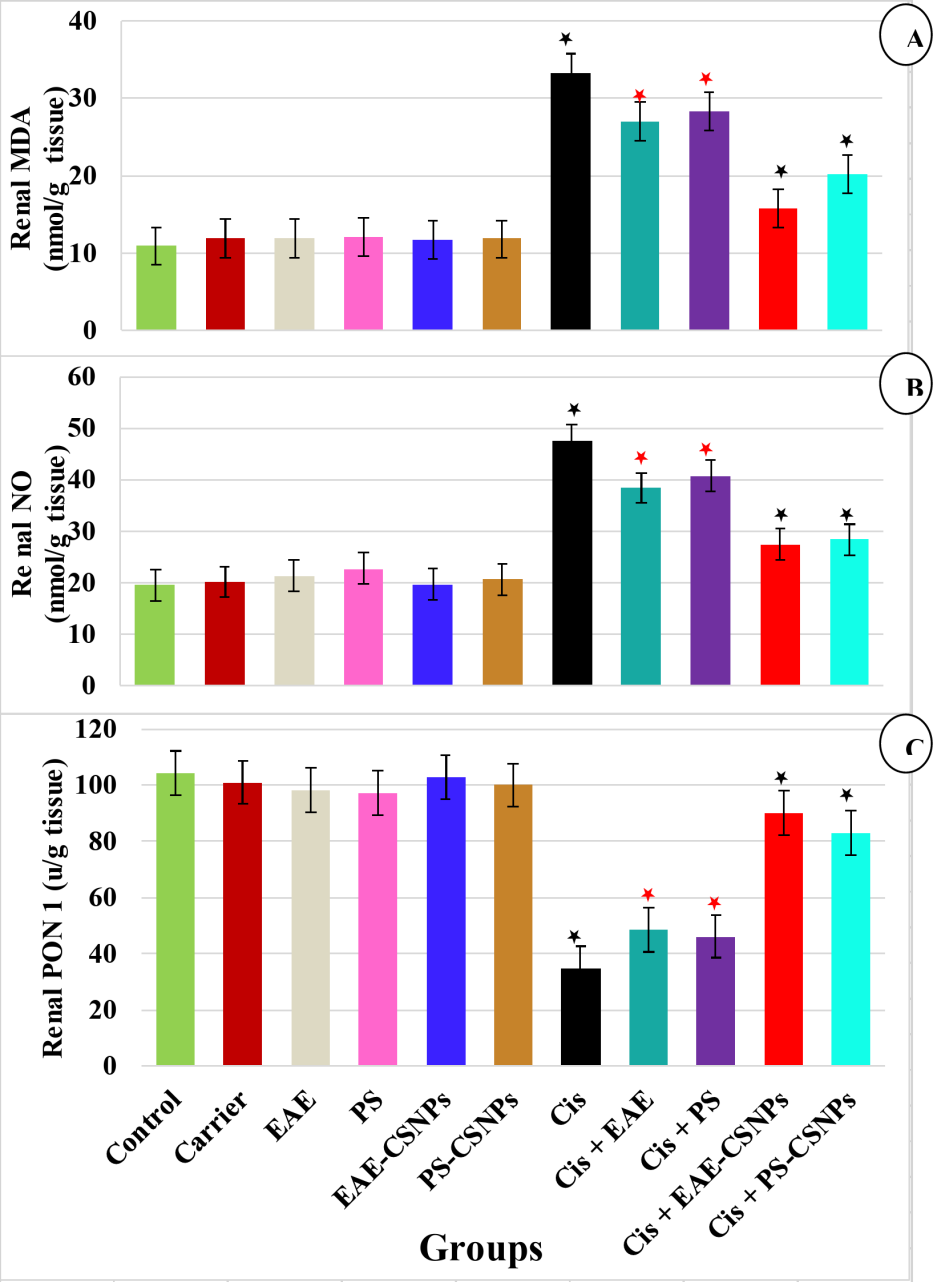
Mean value of (A) Renal MDA (nmol/g tissue), (B) Renal NO (nmol/g tissue) and (C) Renal PON 1(u/g tissue) in different groups. There was no significance difference between the six first groups (control-PS-CSNPs). There was significance difference between Cis group and Cis + EAE and Cis + PS (*p* < 0.05*), while there was high significance between Cis group and Cis + EAE-CSNPs and Cis + PS-CSNPs (*p* < 0.001*). Error bars represent standard error.

In the current study, the administration of CDDP to rats caused nephrotoxicity, as shown by the highly substantial elevation in serum urea and creatinine, renal MDA and NO, with highly significant reduction in creatinine clearance and renal PON 1(*P* = 0.000) compared to the control group. Animals treated orally with EAE or PS after induction of nephrotoxicity showed an observable reduction in serum urea and creatinine, renal MDA and NO levels (*p* < 0.05) and an observable elevation in creatinine clearance and renal PON 1 level (*p* < 0.05) in comparison to the CDDP group. Meanwhile, nephrotoxic rats treated with EAE-CSNPs or PS-CSNPs demonstrated more observable reduction in serum urea and creatinine, renal MDA and NO levels (*p* < 0.001), and highly observable elevation in creatinine clearance and renal PON 1 level (*p* < 0.001) in comparison to the CDDP group.

On the other hand, no significant variations were detected in the biochemical parameters between all groups not treated with CDDP and the control group.

#### Effect of treatments on renal nephrin and podocin, their renal mRNA relative expressions (Fig. 5) and urinary nephrin and podocin (Fig. 6):

**Fig. 5:**
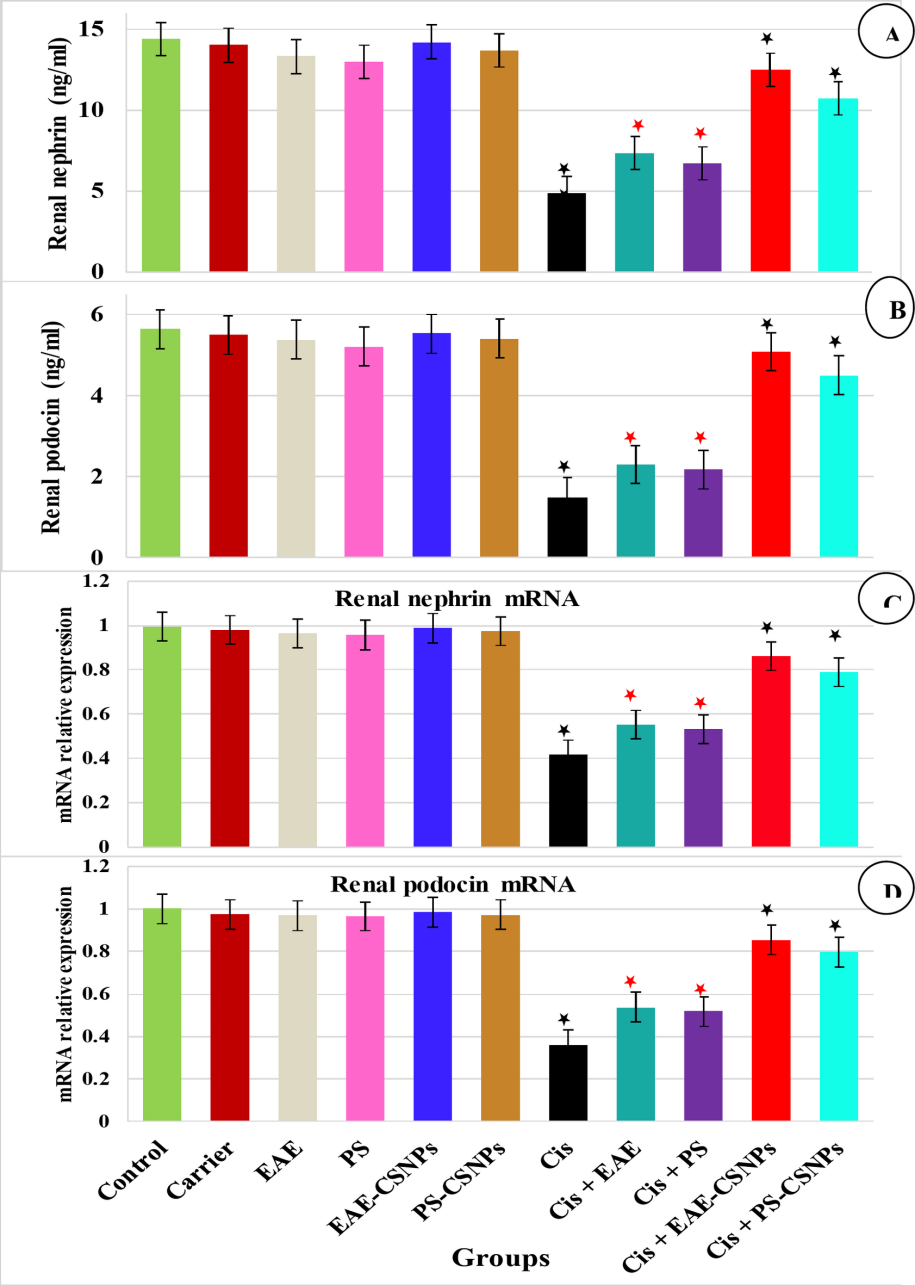
Mean value of (A) Renal nephrin (ng/ml), (B) Renal podocin (ng/ml), (C) Relative expression of renal nephrin mRNA and (D) Relative expression of renal podocin mRNA in different studied groups. There was no significance difference between the six first groups (control-PS-CSNPs). There was significance difference between Cis group and Cis + EAE and Cis + PS (*p* < 0.05*), while there was high significance between Cis group and Cis + EAE-CSNPs and Cis + PS-CSNPs (*p* < 0.001*). Error bars represent standard error.

**Fig. 6:**
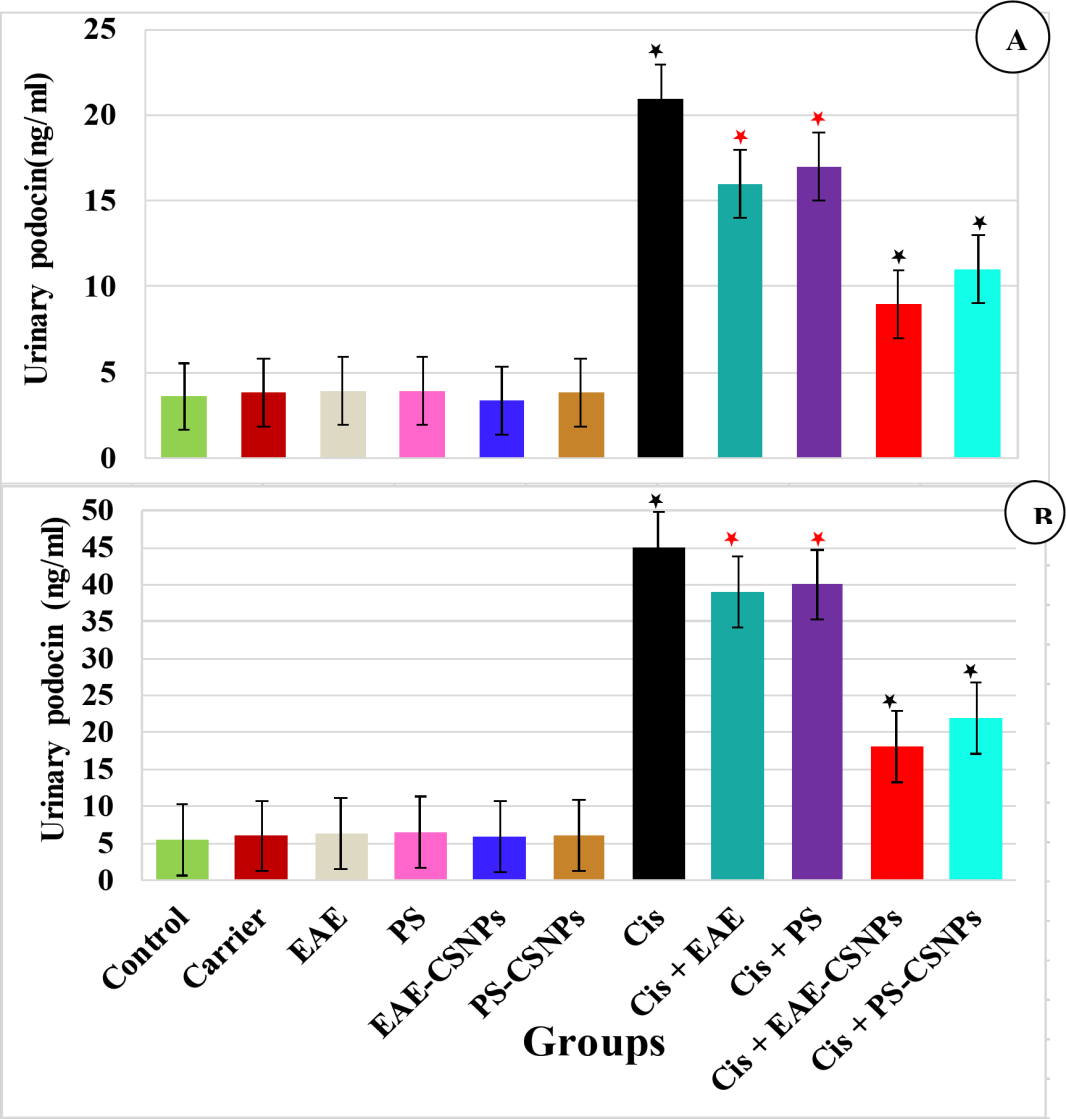
Mean value of (A) Urinary nephrin (ng/ml) and (B) Urinary podocin (ng/ml) in different studied groups. There was no significance difference between the six first groups (control-PS-CSNPs). There was significance difference between Cis group and Cis + EAE and Cis + PS (*p* < 0.05*), while there was high significance between Cis group and Cis + EAE-CSNPs and Cis + PS-CSNPs (*p* < 0.001*). Error bars represent standard error.

Firstly, nephrin and podocin levels in either kidney or urine, in addition to their renal mRNA relative expressions, clarified that there are no significant differences among the normal control, carrier, EAE, PS, EAE-CSNPs and PS-CSNPs groups.

Secondly, the CDDP group showed a highly substantial reduction in renal nephrin and podocin levels and their renal mRNA relative expressions (*P* = 0.000), along with highly significant increase in urinary nephrin and podocin levels (*P* = 0.000) compared to the control group. Meanwhile, rats treated orally with EAE or PS after induction of nephrotoxicity demonstrated an observable increase in renal nephrin and podocin levels and their renal mRNA relative expressions (*P* < 0.05), with a significant decrease in urinary nephrin and podocin (*p* < 0.05) compared to the CDDP group. On the other hand, nephrotoxic rats treated with EAE-CSNPs or PS-CSNPs demonstrated higher improvement, as evidenced by a more observable increase in renal nephrin and podocin levels and their renal mRNA relative expressions (*p* < 0.001), with highly significant decrease in urinary nephrin and podocin (*p* < 0.001) in comparison to the CDDP group.

### Histopathological examination

**Fig. 7:**
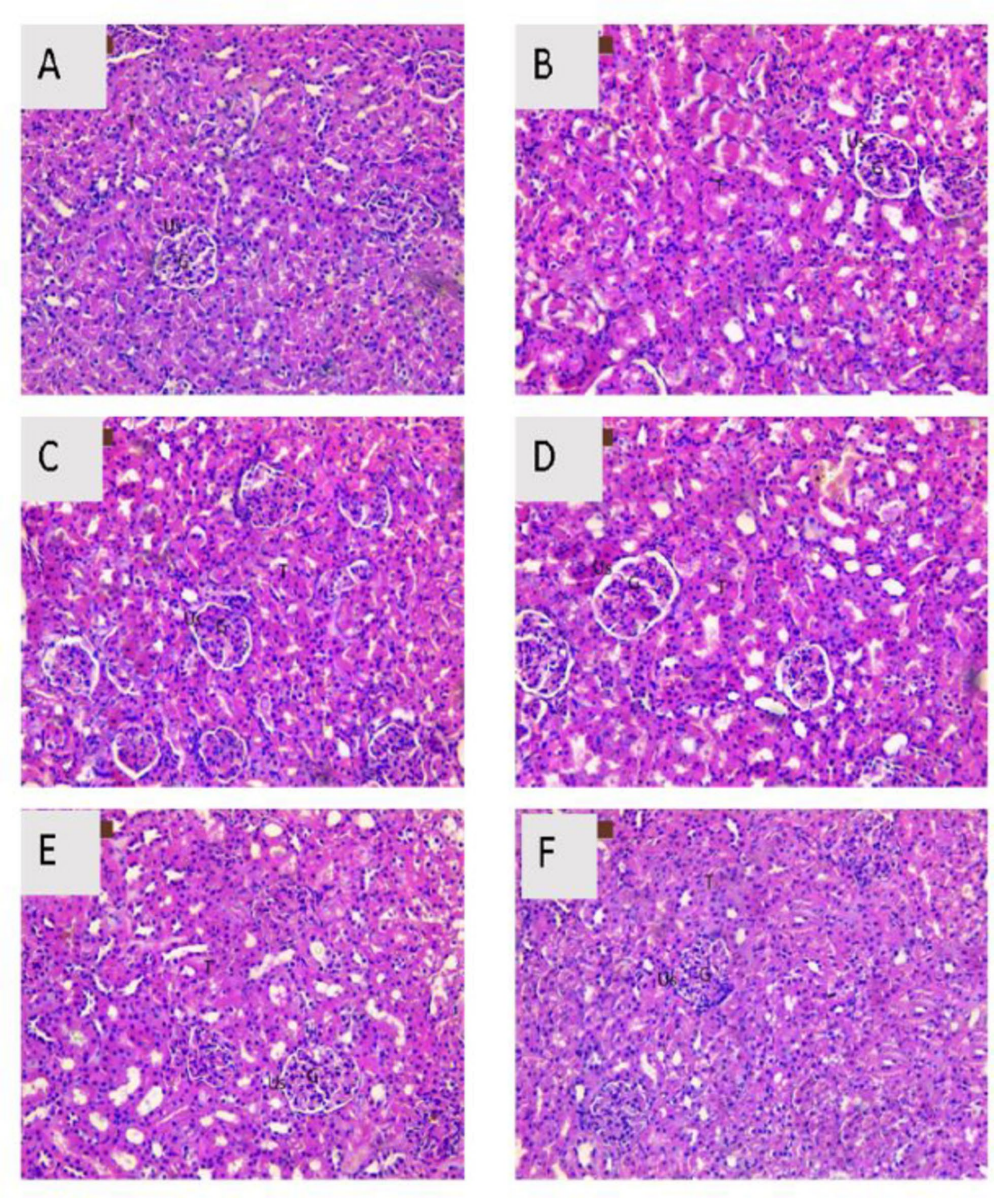
A photomicrograph of H&E stained section from rat kidney of (A) Control group, (B) Carrier group, (C) EAE group, (D) PS group, (E) EAE-CSNPs group and (F) PS-CSNPs group showed normal structure of the glomerulus (G), urinary space (Us) and tubules (T) (Magnification x 200).

**Fig. 8:**
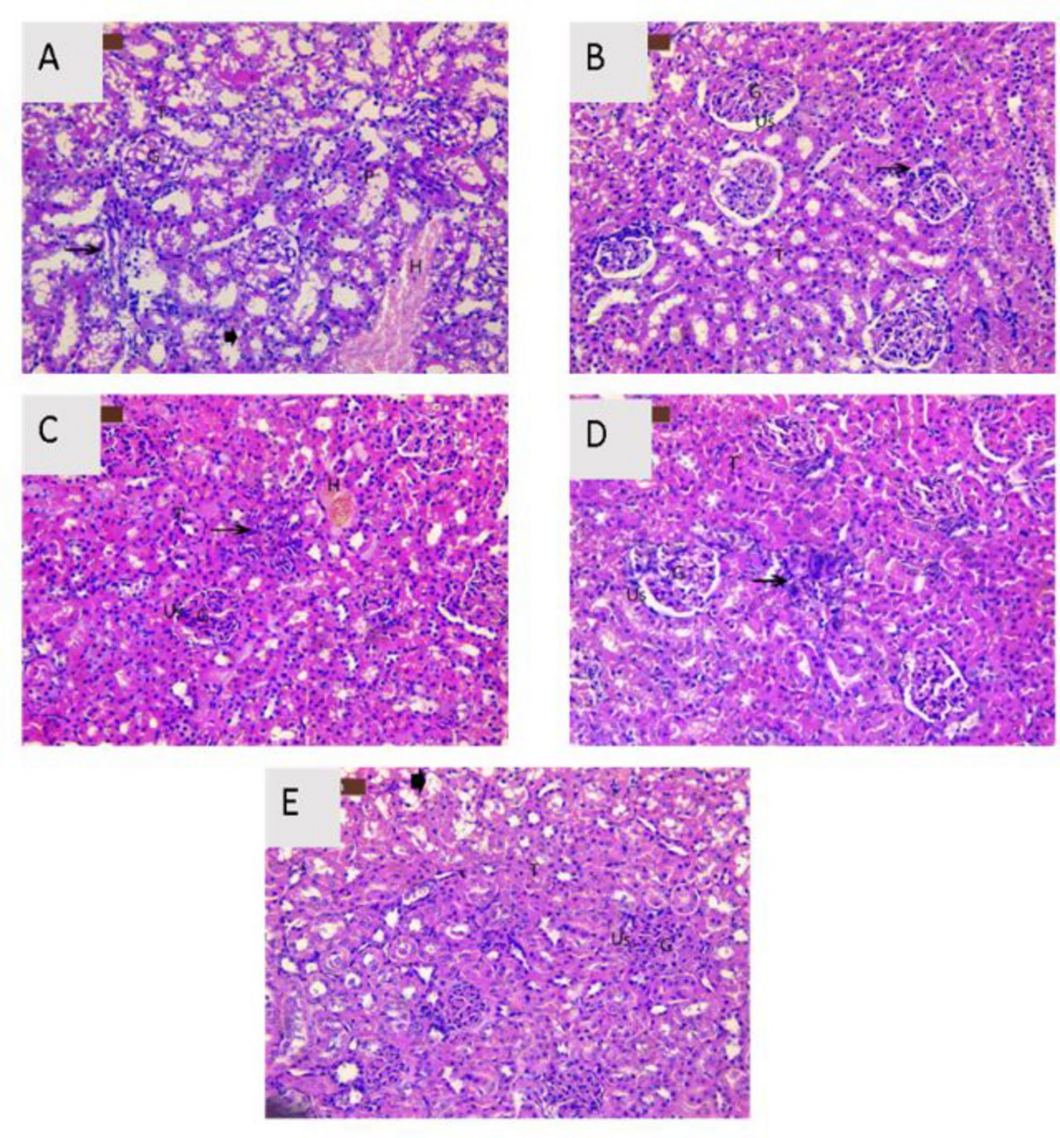
A photomicrograph of H&E stained section from rat kidney of (A) Cis group showed shrunken glomeruli (G) with dilated urinary space (Us), tubular epithelial cell degeneration (T), necrosis associated with interstitial inflammatory cells and interstitial hemorrhage, (B) Cis + EAE group, (C) Cis + PS group showed moderate ameliorative effect with slight atrophy of glomeruli (G), mild dilated urinary space (Us) and mild necrosis with mild inflammatory cells, (D) Cis + EAE-CSNPs group, (E) Cis + PS-CSNPs group showed marked ameliorative effect with slight atrophy of glomeruli (G) and mild dilated urinary space (Us) (Magnification x 200).

Light microscopic images from renal tissues of the control, carrier (CSNPs), EAE, PS, EAE-CSNPs, and PS-CSNPs groups showed normal tubular structures, normal glomeruli architecture, with normal Bowman’s space (Fig. 7) and (Table 3). Histopathological investigation of sections from rat kidneys after two weeks of CDDP injection (CDDP group) displayed impaired renal morphology evidenced by shrunken glomeruli with dilated urinary space, necrosis associated with interstitial inflammatory cells, and interstitial haemorrhage in addition to tubular epithelial cell degeneration with pyknotic nuclei (Fig. 8A) and (Table 4). The kidneys of the (CDDP + EAE) and (CDDP + PS) groups showed less atrophy of glomeruli, less dilated urinary space, and mild necrosis with mild inflammatory cells (Fig. 8, B&C), and (Table 4). Kidneys of the groups received (CDDP + EAE-CSNPs) or (CDDP + PS-CSNPs) showed approximately typical kidney architecture in most of glomerular and Bowman’s space with intact epithelial cells and mild dilated urinary space (Fig. 8, D&E) and (Table 4).

**Table 3:** Semi-quantitative scoring of damage on histopathological analysis of the kidney of control group and groups treated with CSNPs, EAE, PS, EAE-CSNPs or PS-CSNPs.

Groups	Shrunken glomeruli	Dilated urinary space	Tubular degeneration	Interstitial hemorrhage	Interstitial inflammatory	Pyknotic nuclei
Control	-	-	-	-	-	-
Carrier (CSNPs)	-	-	-	-	-	-
EAE	-	-	-	-	-	-
PS	-	-	-	-	-	-
EAE-CSNPs	-	-	-	-	-	-
PS-CSNPs	-	-	-	-	-	-

Note: Histological analysis was done according to four grades: − (nil); + (mild); ++ (moderate) and +++ (severe).

**Table 4:** Semi-quantitative scoring of damage on histopathological analysis of the kidney of Cis group and groups treated with Cis + EAE, Cis + PS, Cis + EAE-CSNPs and Cis + PS-CSNPs.

Groups	Shrunken glomeruli	Dilated urinary space	Tubular degeneration	Interstitial hemorrhage	Interstitial inflammatory	Pyknotic nuclei
Cisplatin (Cis)	++++	+++	++++	+++	+++	+++
(Cis) + EAE	++	++	++	++	++	++
(Cis) + PS	++	++	++	++	++	++
(Cis) + EAE-CSNPs	+	+	+	-	+	+
(Cis) + PS-CSNPs	+	+	+	+	+	+

Note: Histological analysis was done according to four grades: − (nil); + (mild); ++ (moderate); +++ (severe), ++++ (very severe).

## Discussion

Cisplatin, a chemotherapeutic medication, is extensively employed in the management of cancer. Regrettably, CDDP-induced nephrotoxicity is a commonly encountered adverse reaction. The extent of renal impairment triggered by CDDP is contingent upon the dosage and duration of treatment^17^. More than 25% of patients experience acute nephrotoxicity subsequent to CDDP administration. This is attributed to the accumulation of the drug within the cells of the proximal tubules of the kidney, coupled with its deleterious impact on the glomerular filtration rate (GFR)^2^.

In the present study, the higher antioxidant activity observed for EAE than that recorded for PS may be attributed to the high phenolic content in EAE (35.7 mg GAE/ g of dry fraction). In a study performed by Karimi et al.^13^, ethyl acetate was observed to be a selective solvent for the extraction of phenolic compounds either with low or high molecular weights that possess high antioxidant activity. Sahoo et al.^18^ reported that the antioxidant activity of polyphenol components contained in algal extracts could be attributed to their ability to donate hydrogen atoms or electrons to capture the free radicals, and this agreed with the results of the current study.

The ionic gelation technique was selected for the encapsulation of EAE and PS using chitosan as a source for chitosan nanoparticles in this study. TEM analysis displayed that the average particle sizes of the fabricated nanoparticles were as follows: CSNPs > PS-CSNPs > EAE-CSNPs. The decrease in average particle size after incorporation of the two extracts (EAE & PS) could be attributed to the highest electrostatic interaction between the extract, chitosan polymer chains, and TPP^19^. DLS analysis also revealed that the CSNPs, EAE-CSNPs, and PS-CSNPs were observed at larger diameters in comparison to particle size measurements by TEM, which is due to the formation of a hydration shell during DLS measurements^20^. In addition, results of zeta potentials for chitosan nanoparticles solution either alone or loaded with EAE or PS demonstrated a positive zeta potential with stable suspensions in a decreasing order as follows: EAE-CSNPs > PS-CSNPs > CSNPs. Kriegseis et al.^21^reported that an increase in zeta potential value increases suspension stability. The addition of bioactive components to solutions of chitosan affects the surface positive charge on chitosan, which leads to a change in the stability of the nanoparticles^22^. The literature has revealed that a minimum value of zeta potential (± 30 mV) is required for a stable suspension^21^.

In the current study, it was reported that all the synthesized nanoparticles were homogenous, as illustrated by the PDI value, which was less than 0.5, confirming the effective preparation of well stabilized chitosan nanoparticles in nonaggregate form. A PDI value between 0 and 0.5 indicates homogeneous particles, but values larger than 0.5 suggest heterogeneous particle sizes^23^. From the previous results, it was observed that the most stable suspension was EAE-CSNPs, followed by PS-CSNPs, then CSNPs. Many approaches with selective targeting to injured cells to relieve the toxic effects of a chemotherapeutic drug have been studied^23^. This study was performed to detect the possible ameliorative effect of nanoparticles loaded with EAE or PS (EAE-CSNPs & PS-CSNPs) compared to EAE or PS alone on CDDP-induced nephrotoxicity and podocyturia.

In the present study, the CDDP group showed a highly observable elevation in serum urea and creatinine and a highly substantial reduction in creatinine clearance. These results were similar to those of Kabel et al.^24^ and Gilani et al.^25^, who attributed the elevation in serum urea and creatinine and the decrease in creatinine clearance to the reduction in renal blood flow, GFR, as well as tubular necrosis. The glomerular basement membrane is covered by adjacent podocytes in order to avoid the loss of proteins in the urine. Podocyte detachment is the mechanism by which proteinuria appears and glomerular filtration rate decreases. Hence, the significant decrease in serum albumin may be interpreted by podocyte loss through the mechanism in which urokinase-type plasminogen activator receptor (uPAR) mediates foot process effacement and proteinuria by interaction with integrin αVβ3, leading to integrin activation and podocyte damage^26^.

On the other hand, this study recorded an observable reduction in serum urea and creatinine, with an observable elevation in creatinine clearance in all treated nephrotoxic groups, with more substantial changes in groups treated with loaded nanoparticles (EAE-CSNPs & PS-CSNPs) compared to the CDDP group, indicating significant improvement using nanoparticles. Mikušová and Mikuš^22^ reported that cationic nanoparticles interact with negatively charged heparan sulfate proteoglycans in the glomerular basement membrane, enhancing drug specificity to the kidney, and this result supports the best results gained using chitosan nanoparticles in the present study. In comparison, EAE-CSNPs with PS-CSNPs, better improvement was achieved through EAE-CSNPs and this may be attributed to the highest antioxidant activity for EAE in the current research.

Cisplatin administration in adult male albino rats revealed an observable increase in renal MDA and NO, with an observable reduction in renal PON 1, and marked tubular necrosis, as demonstrated by renal histopathological examination. Many investigations had suggested that oxidative stress and free radicals were complicated in cisplatin nephrotoxicity due to the reduction in the activities of some renal antioxidant enzymes, such as PON 1 and superoxide dismutase (SOD), and the elevation of renal MDA and NO levels^26^. It has been recorded that CDDP can lead to changes in the electron transport chain; consequently, ROS formation and oxidative stress were recognized and linked to CDDP related complications^27^. NO can modify mitochondrial membrane potential by opening the transition pores of mitochondria and releasing cytochrome C, which stimulates the caspase 3 activation, leading to cell death^28^. Omar et al.^29^ stated that CDDP administration significantly increased the lipid peroxidation level as MDA, elevated the NO level, and caused a reduction in PON1, proposing an elevation in the level of oxidative stress. Their results agreed with those of the present study. Administration of EAE or PS either alone or loaded on chitosan nanoparticles to nephrotoxic rats, showed a substantial reduction in renal MDA and NO and an observable rise in renal PON 1, with more noticeable changes in the case of loaded nanoparticles compared to the CDDP group, indicating better results obtained using nanotechnology. These results may be attributed to the greatest level of antioxidants present in the brown alga *T. triquetra*extracts. Abd El Hafez^30^ reported that the radical scavenging activity of the methanolic extract of *T. triquetra* (at a concentration of 1000 µg/ml, IC50 value of 671.50 µg/ml, with 74.46% inhibition) was more efficient than that of the green alga *Ulva prolifera* and the red alga *Hypnea cornuta*. de Melo et al.^31^reported that fucoidan exhibited excellent antioxidant capacities and can be used in the management of oxidative stress. Treatment using antioxidants such as plant extracts and vitamin E before and after CDDP administration could prevent the reduction of the renal antioxidant system^12^. These results were in agreement with those obtained from the current study. In the CDDP group, renal nephrin and podocin and their renal mRNA gene expressions were highly reduced, while urinary nephrin and podocin were significantly increased compared to the control group, indicating progressive podocyturia. The podocyte is an important component of the GFB, and its injury causes numerous glomerular diseases, which can result in foot process fusion and detachment of the cell from the GBM, thus leading to proteinuria and glomerulosclerosis^32^. Barutta et al.^33^reported that the molecular processes that cause glomerular diseases are not completely clear, although increasing evidence suggests an important role for podocytes in permeability changes at the GFB. Two mechanisms were suggested for the downregulation of nephrin mRNA in the CDDP group. One is the alteration of the structure of filtration slits and foot processes, while the other is podocyturia^34^.

Podocytes can react by specific receptors on their surface to a group of inflammatory mediators, including ROS, complement products produced during glomerular inflammatory states, and cytokines. Podocytes may experience hemodynamic difficulties as a result of their downstream location from the GBM, which subjects them to ongoing stress that is exacerbated in settings of hyperfiltration^35^. Additionally, hemodynamic and architectural podocytopathic alterations can be caused by hyperfiltration brought by a reduction in the number of functional nephrons^35^. Podocyte activation by hemodynamic and/or immunological factors eventually results in protein unfolding abnormalities, ROS release, endoplasmic reticulum stress, and mitochondrial damage. The podocyte stress impacts the integrity of the slit diaphragm and cytoskeleton, helping in cell damage, detachment, and death^26^. Structure changes in nephrin and podocin can cause severe proteinuria^36^. Lu et al.^32^reported that experimental models of glomerulonephritis have demonstrated an association between changes in nephrin or podocin expressions, and this agrees with the results of the current study. Sekulic and Sekulic^37^ reported that nephrin and podocin proteins could be measured in the urine with a significant percentage in diabetics, and this highlights the screening value of these podocyte specific proteins as biomarkers for podocyturia and nephrotoxicity. In the present study, all the groups injected with CDDP and then treated with *T. triquetra*extracts either alone or loaded on nanoparticles revealed an observable increase in renal nephrin, its mRNA, renal podocin, and its mRNA, with a significant decrease in urinary nephrin and urinary podocin; however, the loaded nanoparticles showed more significant changes compared to the CDDP group, indicating a more better ameliorative effect and higher improvement in kidney functions using nanoencapsulated extracts than that using unloaded extracts (EAE & PS). This ameliorative effect may be attributed to inhibiting the synthesis of a urokinase-type plasminogen activator receptor^26^. As a result of this inhibition, the activation of αVβ3 integrins was inhibited, and podocyte detachment was reduced^26^. All previous results were compatible with the results of the histopathological examination in all studied groups.

In this study, the histopathological examination of the CDDP group revealed types of changes such as impaired renal morphology throughout tubular epithelial cell degeneration, shrunken glomeruli with markedly dilated urinary space, necrosis associated with inflammatory cells, and interstitial hemorrhage. Abnormalities in the function and structure of renal tissue are supposed to be related to oxidative stress and inflammation that are triggered by chemokines such as tumor necrosis factor. Many investigations have reported that apoptosis is complicated by cisplatin nephrotoxicity^38,39^. Nephrotoxic rats treated with loaded nanoparticles revealed remarkable ameliorative effect, with almost normal architecture of the kidney in most of the glomerular and Bowman’s space with mild interstitial inflammatory cells, while those treated with unloaded extracts demonstrated moderate ameliorative effects with mild dilated urinary spaces, slight atrophy of glomeruli, and mild necrosis of tubules. On the other hand, no significant alterations were detected in all studied biochemical parameters between all groups not treated with CDDP and the control group, as evidenced by histopathological examinations that illustrated normal tubular structures, normal glomeruli architecture, with normal Bowman’s space. This observation indicates that *T. triquetra*extracts as well as chitosan nanoparticles are safe and non-toxic compounds. Michalak and Chojnacka^39^ reported that products derived from algae are safe for humans, animals, and plants. Their results were in agreement with those of the present study. Hence, they can be used in modern agriculture and in food, cosmetic, and pharmaceutical industries.

## Materials and methods

### Chemicals

Cisplatin, Folin-Ciocalteu reagent, Gallic acid, 2,2-Diphenyl-1-picrylhydrazyl (DPPH), Ascorbic acid, Chitosan (CS)(Mw: 190–310 KD, DD: 75–85%), and Sodium tripolyphosphate (STPP) were ordered from Sigma-Aldrich Chemical Company (St. Louis, USA).

### Brown alga collection and cleaning

Fresh samples of the brown alga *Turbinaria triquetra*(J. Agardh) Kützing were collected and identified by prof. Dr. Nihal Galal El-Din Shams El-Din, (Professor in Hydrobiology Lab, National Institute of Oceanography and Fisheries (NIOF), Cairo, Egypt) in autumn (2017). The collection was complied with relevant institutional, national, and international guidelines and legislation. Samples were identified using the catalogue of Braun and Guiry^40^. The taxonomic position of the species was updated according to the site of Algae Base^41^.

Location, Hurghada, Red Sea, Egypt, in front of the National Institute of Oceanography and Fisheries (NIOF) at 31°13.3`N latitude and 29°53.10`E longitude.

The alga samples were entirely handpicked and washed at the site of collection with seawater to eliminate the adhered sediments, then placed in polyethylene bags. The samples were kept at 4 °C. At the laboratory, the samples of the alga were rinsed through tap water to eliminate the epiphytes. *Turbinaria triquetra* belongs to the Class Phaeophyceae, Order Fucales, Family Sargassaceae. A voucher specimen (M.AL.1) was deposited in the herbarium of the National Research Centre, Cairo, Egypt (CAIRC).

About 1 kg of the samples was dried in the air and shade at nearly 25 °C. Then, the algal sample was milled to a fine powder for further analysis.

### Preparation of ethyl acetate fraction

A weight of 450 g of *T. triquetra* powder was extracted by maceration in 70% methanol, then sonication for 20 min. The extract was filtrated, and the previous steps were repeated to exhaustion. The combined extracts were evaporated at 45 ^o^C to dryness under reduced pressure, yielding a crude extract of 58.27 g. The crude extract was subjected to re-dissolving in distilled water and partitioning against ethyl acetate. The previous steps were repeated several times, and the ethyl acetate layers were separated, collected, and evaporated till dryness^42^, yielding a reddish brown sticky fraction (EAE, 3.86 g).

### Preparation of polysaccharide

The polysaccharide (PS) was extracted by the boiling water technique and deproteinized by the sevage method as previously described by Mettwally et al.^43^, yielding polysaccharide (PS, 11.36 g).

### Determination of antioxidant activity

The antioxidant activities were measured by the DPPH assay method, as previously mentioned by Chang et al.^44^. A standard curve using ascorbic acid was prepared, and the antioxidant activity of EAE was calculated and then compared to that of PS.

### Determination of total phenolic content

The total content of phenolics in EAE was determined using the Folin-Ciocalteu Method^42^. A standard curve using gallic acid was depicted to determine the total content of phenolics in triplicate as mg gallic acid equivalent (GAE)/g dry fraction.

###  Encapsulation of EAE using chitosan using ionic gelation

The nanoparticles were prepared by ionic gelation of CS with sodium tripolyphosphate (STPP), as previously mentioned by Antoniou et al.^45^, with a few modifications. In brief, 0.2% (w/v) CS solution was prepared in 1% (v/v) aqueous glacial acetic acid. In parallel, EAE, 0.7 mg/ml, was prepared in STPP aqueous solution (0.1% w/v), and then added to CS solution dropwise by ratio (1:2) to obtain CSNPs loaded with EAE (EAE-CSNPs). On the other hand, CSNPs and CSNPs loaded with PS (PS-CSNPs) were prepared as previously mentioned by Abdel-Aal et al.^16^.

### Microstructure characterizations

The morphological characteristics of EAE-CSNPs were studied by a high-performance imaging transmission electron microscopy (TEM) machine (JEOLH-7650, Tokyo, Japan) with an acceleration voltage operating at 200 kV^45^. Additionally, the hydrodynamic size and zeta-potential of these nanoparticles were determined at 25 ^o^C on a Zetasizer (Malvern Instruments, Worcestershire, UK) based on the principle of dynamic light scattering (DLS)^46^. The morphological characteristics of CSNPs and PS-CSNPs observed in the previous study by Abdel-Aal et al.^16^ were compared to those of EAE-CSNPs observed in the current study.

### Animal ethics

Male Wistar rats (8–10 weeks old; weighing 130 ± 20 g) were bought from the Animal House of the National Research Centre (NRC), Dokki, Egypt. The rats were put in clean polypropylene cages under controlled temperature (25 ± 2ºC) and humidity (55%), with a 12 h light/dark cycle. The rats were given water and a standard diet ad libitum during the experiment. The experiments were completed in agreement with protocols and guidelines approved by the Institutional Animal Ethics Committee (Code No: Sci-Ch-M-2021-99: dated 24.05.2021), Mansoura University, Mansoura, Egypt and in accordance with ARRIVE guidelines.

### In vivo experimental design

After 14 days of acclimatization period, 66 rats were equally and randomly distributed into 11 groups (6 rats in each). In the study, the dose of CDDP was determined according to Mapuskar et al.^46^, while the dose of CSNPs, EAE, PS, EAE-CSNPs, and PS-CSNPs was determined according to Rao et al.^47^. Group 1 (Control untreated group): Normal rats left without any treatment. Group 2 (Carrier group): Normal rats received CSNPs as a carrier .Group 3 (EAE group): Normal rats received EAE. Group 4 (PS group): Normal rats received PS. Group 5 (EAE-CSNPs group): Normal rats received EAE-CSNPs. Group 6 (PS-CSNPs group): Normal rats received PS-CSNPs. Group 7 (CDDP group): Normal rats were administered intraperitoneally (i.p) with a single dose of CDDP (10 mg/kg bw, two weeks before starting the experiment to induce nephrotoxicity). Group 8 (CDDP + EAE group): Normal rats were administered with CDDP, and then received EAE. Group 9 (CDDP + PS group): Normal rats were administered with CDDP, then received PS. Group 10 (CDDP + EAE-CSNPs group): Normal rats were administered with CDDP, then received EAE-CSNPs Group 11 (CDDP + PS-CSNPs group): Normal rats were administered with CDDP, then received PS-CSNPs. Groups from 2 to 6 received their substances orally (100 mg/kg bw, day after day for 4 weeks). Groups from 8 to 11 were administered CDDP intraperitoneally (i.p) with a single dose (10 mg/kg bw, till reaching nephrotoxicity) and then received their substances orally (100 mg/kg bw, day after day for 4 weeks).

### **Urine**,** blood and tissue sampling**

The rats were fasted for 24 h. when the study (4 weeks) ended, and then urine samples were collected in sterile containers, centrifuged for 10 min at 5000 x g, and finally frozen at −20 °C. Samples of blood were withdrawn from the retro-orbital venous plexus, placed in plain tubes, and then centrifuged for 10 min at 4000 x g for serum separation. Decapitation of the rats performed as a type of euthanasia that maintains the tissue architecture, then kidneys were removed and frozen at −80°C until homogenization^48^.

### Investigations of the biochemical parameters

Serum urea^48^as well as creatinine in serum and urine^49^ were detected spectrophotometrically using commercial kits (Bio-diagnostic Co., Egypt). The formula below was used to determine creatinine clearance:

Creatinine clearance (ml/min/kg bw) =$\:\frac{\text{m}\text{g}\:\:\text{c}\text{r}\text{e}\text{a}\text{t}\text{i}\text{n}\text{i}\text{n}\text{e}/\text{d}\text{l}\:\:\text{u}\text{r}\text{i}\text{n}\text{e}\:\:\times\:\:\:\text{m}\text{l}\:\:\text{u}\text{r}\text{i}\text{n}\text{e}\:\:24\:\:\text{h}\text{r}}{\text{m}\text{g}\:\:\text{c}\text{r}\text{e}\text{a}\text{t}\text{i}\text{n}\text{i}\text{n}\text{e}/\text{d}\text{l}\:\:\text{s}\text{e}\text{r}\text{u}\text{m}\:\:\times\:\:\:1440}$

The frozen kidney tissues were cut into small parts and placed in phosphate-buffered saline at pH7.4 (1:5 w/v) for homogenization and centrifuged at 4 °C and 10,000 rpm for 15 min. The supernatant was removed after centrifugation for measuring malondialdehyde (MDA) by the method of Islayem et al.^50^, nitric oxide (NO) as nitrate according to Palm et al.^51^, and paraoxonase 1 (PON 1) using the method of Richter et al.^52^. The levels of nephrin and podocin in the renal tissues and urine of rats were quantified using commercially available ELISA Kits obtained from Wuhan Fine Biotech Co., Ltd. (Wuhan, China). Genes of nephrin (NPHS1) (NM_022628.1) and podocin (NPHS2) (NM_130828.3) were studied relative to β-actin gene (Actb) (XM_039089807.1). Renal nephrin and podocin mRNA levels were determined using a total RNA extraction kit (Invitrogen, Germany), cDNA synthesis kit (MBI Fermentas, Germany), and SYBR Green qPCR kit (Qiagen GmbH Germany). The primers sequence used are shown in (Table 5). The levels of relative mRNA expression were normalized to those of β-actin. Each PCR experiment was repeated independently at least three times.

**Table 5:** Primer list (F , forward primer; R , reverse primer).

Primer name	Sequence (5’ to 3’)
Rat-nephrin-F	TTCACAACACCCGTTTCCTA
Rat-nephrin-R	TTGGTCATCCCAGATGTCAG
Rat-podocin-F	GAGCATTGCCCAAGATGTAAA
Rat-podocin-R	AGTTCTCTCCACTTTGATGCC
Rat-β-actin-F	ATGTGGCTGAGGACTTTGATT
Rat-β-actin-R	ATCTATGCCGTGGATACTTGG

### Histopathological examination

The kidneys have been removed and fixed in 10% neutral buffered formalin-saline at room temperature. Tissues have been handled for paraffin embedding, serial sectioning, and stained with hematoxylin and eosin (H&E) to assess the kidney morphology in each group. Morphometric measurements were performed using a light microscope (Leica DM3000 LED, Cambridge, England)^53^. Ten fields were examined from each kidney tissue, and the degree of kidney damage was evaluated. Each field was examined and assigned for damage severity [none (-), mild (+), moderate (++) and severe (+++)].

### Statistical analysis

The data were analyzed by one-way ANOVA. If there was significance between groups, Tukey’s test was performed, and the data were represented as mean ± standard error (SE). Statistical analysis was accomplished by SPSS software (version 22.0, IBM Corp., Chicago, USA, 2013). P value < 0.05 was recorded as statistically significant, and P value < 0.001 was recorded as highly significant, otherwise it is non-significant.

## Conclusion

The results of the current study concluded that nanoencapsulation using chitosan nanoparticles (EAE-CSNPs and PS-CSNPs) has better ameliorative effects against podocyturia and CDDP nephrotoxicity than extracts (EAE and PS) do alone in rats. The present study offers a new therapeutic strategy for attenuating podocyturia and CDDP nephrotoxicity.

## Data Availability

All data generated or analyzed during this study are included in the manuscript.
